# A Hospital Scandal

**Published:** 1887-08-06

**Authors:** 


### "A HOSPITAL SCANDAL."

Every now and again the daily papers publish stories of alleged inhumanity on the part of hospitals which come upon the public with a shock of painful surprise. When a mother, who is usually regarded as the very incarnation of all that is tender and loving in human nature, behaves towards her own offspring with deliberate and cold-blooded cruelty, the indignation of a generous people reaches a pitch at which self-restraint is difficult. And similarly, if a hospital, which is the natural mother of all the sick and disabled in its neighbourhood, gives unmistakable evidence of indifference and heartless want of sympathy with suffering and pain, the public look upon such a hospital with stern displeasure. A few days ago the *Pall Mall Gazette* published a letter from Mr. Thomas Moran, a working-man, giving a detailed story of what to an uninstructed person looks like a profound failure on the part of hospital officials to understand the nature of their own duties and responsibilities. The facts as related by Mr. Moran are these: William Groves, a tram-car driver, of 6, Mendip Road, Wandsworth, was seen to totter as if about to fall. He did not, however, fall, as assistance was at hand. Groves, it appears, had been on the funds of the sick-club of the Tram-car Company for a fortnight previously, and had vomited a large quantity of blood, whereby he had been made very weak. When the faintness came on he was at once taken to his own doctor, presumably the doctor of the sick-club. After waiting for some time he was examined by the doctor, who advised his removal to St. Thomas's Hospital, stating that he was suffering from epileptic convulsions. When Groves reached St. Thomas's he was there again medically examined, and instead of being taken into the wards, was endeavoured to be transferred to the Lambeth Infirmary—that is, from the care of the hospital to that of the Poor-law authorities. After much discussion, the poor man was taken to Lambeth, and here again admittance was declared to be impossible, until a duly signed order could be produced. Upon this order being sought, it was refused, on the ground that Groves did not belong to the parish. However, when the relieving officer, after being vigorously remonstrated with, had seen the patient, he at once made out the necessary order, and Groves was immediately admitted, and, by the doctor's orders, put to bed. Moran adds: "Surely some one is responsible for this tom-fooling. . . . Such a case as this is a scandal to any country. . . . I cannot explain to you the man's feelings, but anyone suffering acutely from heart-disease may know. . . . I write this as a protest against the way this man was treated." It may be added that if the man really was suffering from heart-disease, and had recently lost three pints of blood as stated, he might have died during this protracted effort to find a lodgment in a hospital.

On the first blush of the case every hospital and infirmary official seems to have behaved with gross stupidity and inhumanity. But it must be remembered that only one side has been heard. It may further be pointed out, that although hospitals exist for the relief of suffering, they can only be carried on under a system of carefully defined regulations. Although accidents and dangerous medical cases occurring suddenly are usually received at once, yet their doors do not stand open night and day for the admittance and retention of any and every person who may choose to apply. If they did, their wards would speedily be crowded with all sorts of persons who have no claim whatever upon public or private charity. The initial mistake seems to us to have been made by Groves' own medical attendant. Why did he send the patient to the hospital instead of to his own home? He knew, or ought to have known, this case was ineligible for admission to a general hospital. Similar "Hospital Scandals" would be avoided if every doctor, and indeed every household, possessed a copy of "Help in Sickness,"* a little book where the rules governing the admission of cases to all medical institutions are classified in a form easy of reference. Moran, the *Pall Mall's* correspondent, says he presented the "doctor's order" at the hospital, and in spite of this "order" the patient was refused admittance. But the public ought to be aware that though a certificate from a medical man in suitable cases is usually a passport to admittance, yet no private medical man has authority to write an "order" for admission to a hospital, any more than has the postman or the lamplighter. If Groves had a home of his own, and belonged to a sick-club to which a doctor was attached, the natural and proper thing to do was to have had him removed there. No hospital, it must be remembered, is largely a refuge for casual cases of sickness which can be treated better at home.

It is further to be borne in mind that Groves was not sent away from St. Thomas's without

* "Help in Sickness," price 1s. 6d. London: Kegan Paul, Trench & Co.

examination. On the contrary, he was at once taken in, and, so far as appears, attended to with the utmost kindness. We may assume also that the medical officer who examined him did not consider him a suitable case for the hospital, seeing that he was certified as suffering from epileptic convulsions, and that epileptics are specially excluded by the rules of most, if not all, general hospitals; nor did he apparently see any special risk connected with his removal to the infirmary. This view appears to have been corroborated by the actual facts, for, so far as present information shows, Groves has come to no serious harm by his removal. Pending further light, therefore, there seems to be no evidence which will substantiate a charge of unkindness or neglect of duty, at any rate against the officials of St. Thomas's Hospital. That there was some delay in finding the poor man a resting-place was unfortunate, but it was due to the routine—the necessary routine—which has usually to be gone through on such occasions. All the delay might have been avoided by sending him direct to his own home, or retaining him for an hour in the doctor's waiting-room until he had had time to recover his strength a little.

We call attention to this case for a twofold reason. Our desire in the first place is to warn the public not to attach too much importance to highly-coloured letters and reports which may appear from time to time in the newspapers. It should be a standing rule, never to be broken, that before a decision is come to on the subject, any accused institution or individual shall be heard in his own defence. Even the old Roman law condemned no man unheard; neither should public opinion. But we wish still more to point out that if the metropolis had possessed an efficient ambulance system, such a case as that of Groves' could by no possibility have occurred. A system of ambulance organised as in Liverpool and other places, would at once make all the metropolitan hospitals and infirmaries immediately available for any accidental or other emergency however sudden. The appearance of a recognised public ambulance at the hospital gates would be an order for immediate admission which no official could or would question. If the public wish to have the comforting assurance that any citizen who is suddenly taken ill in the streets will not be passed on from one inhospitable refuge to another, but be promptly placed in a home where all that medical science and nursing skill can do will be immediately and ungrudgingly available, let it without any delay establish on a permanent basis a complete system of ambulance for every part of the metropolis. Then, and not till then, shall we cease to see, what we so often see now, "Another Hospital Scandal" as an alarming heading to a newspaper paragraph.

